# A Lightweight and Efficient Multimodal Feature Fusion Network for Bearing Fault Diagnosis in Industrial Applications

**DOI:** 10.3390/s24227139

**Published:** 2024-11-06

**Authors:** Chaoquan Mo, Ke Huang, Wenhan Li, Kaibo Xu

**Affiliations:** College of Mechanical & Electrical Engineering, Wenzhou University, Wenzhou 325035, China; 22451439021@stu.wzu.edu.cn (C.M.); 23451145017@stu.wzu.edu.cn (W.L.); 23461146094@stu.wzu.edu.cn (K.X.)

**Keywords:** mechanical equipment fault diagnosis, parallel time–frequency dual-stream, multimodal feature fusion, convolutional attention mechanism, graphical user interface

## Abstract

To address the issues of single-structured feature input channels, insufficient feature learning capabilities in noisy environments, and large model parameter sizes in intelligent diagnostic models for mechanical equipment, a lightweight and efficient multimodal feature fusion convolutional neural network (LEMFN) method is proposed. Compared with existing models, LEMFN captures rich fault features at multiple scales by combining time-domain and frequency-domain signals, thereby enhancing the model’s robustness to noise and improving data adaptability under varying operating conditions. Additionally, the convolutional block attention module (CBAM) and random overlapping sampling technology (ROST) are introduced, and through a feature fusion strategy, the accurate diagnosis of mechanical equipment faults is achieved. Experimental results demonstrate that the proposed method not only possesses high diagnostic accuracy and rapid convergence but also exhibits strong robustness in noisy environments. Finally, a graphical user interface (GUI)-based mechanical equipment fault detection system was developed to promote the practical application of intelligent fault diagnosis in mechanical equipment.

## 1. Introduction

With the rapid development of modern industry and science and technology, highly precise and integrated industrial equipment has entered various fields, increasing the risk rate of mechanical equipment failures [1]. Mechanical transmission systems in equipment such as vehicles, wind turbines, chemical processing machinery, and construction machinery often operate under complex and harsh conditions, including heavy loads, fatigue, corrosion, and high temperatures. As a result, core components in these systems inevitably suffer varying degrees of damage. For example, main shafts, gears, and bearings frequently experience wear, breakage, tooth fractures, root cracks, spalling, welding, and scoring. Once a failure occurs in a mechanical transmission system, it can lead to significant economic losses and casualties. Therefore, extensive research on the diagnosis of damage faults in the main shafts, gears, and bearings of mechanical transmission systems has been carried out both domestically and internationally [2]. Numerous artificial intelligence diagnostic models have been proposed, such as Bayesian theory, fuzzy set theory, D-S evidence reasoning, genetic algorithms, cluster analysis, support vector machines (SVMs), extreme learning machines (ELMs), convolutional neural networks (CNNs), and Transformers. These provide basic tools for mechanical equipment fault diagnosis and have become a hotspot and promising research direction in the field of mechanical fault diagnosis [3,4,5]. The application of artificial intelligence diagnostic models can alleviate the problem of over-reliance on specialized technical personnel and diagnostic experts, achieving efficient and reliable online diagnostics. Their role and benefits are increasingly evident, covering a broader range, and are widely recognized by many researchers [6,7,8]. However, due to the presence of operational frequency vibrations, electrical noise, hydraulic pulsation noise, modulation, and noise interference in actual production, the characteristic information monitored online is weak, and the robustness of feature extraction methods is poor. Therefore, new solutions for mechanical equipment fault diagnosis remain a hotspot and challenge in domestic and international research [9].

Currently, mainstream architectures in deep learning for fault diagnosis include recurrent neural networks (RNNs) [10], convolutional neural networks (CNNs) [11], and Transformer neural networks [12]. While RNNs can handle sequential data, they cannot perform parallel computing and are not suitable for training large-scale datasets. Although long short-term memory networks (LSTMs) [13], as variants of RNNs, alleviate long-range dependency problems to some extent, they require the manual extraction of time–frequency domain features and lack inherent feature extraction capabilities. Traditional Transformer models often adopt hierarchical frameworks, making it difficult to integrate feature information between different layers, weakening the ability to learn local features. Additionally, Transformer models have a large parameter scale, requiring substantial computational resources, leading to low work efficiency and high computational costs, presenting significant challenges in deployment and application within the industrial sector [14]. Compared to RNN models and Transformer models, CNN models adopt designs such as local connections, weight sharing, and spatial pooling, making the model lighter, training faster, and feature learning superior, thus being more widely applicable.

In recent years, researchers have made significant progress in applying deep learning to mechanical fault diagnosis [15]. Deep learning can automatically extract features from datasets, reducing reliance on expert prior knowledge. Deep convolutional neural networks as deep learning models have the capability to handle periodic signals, capable of processing known grid-like one-dimensional time-series data or two-dimensional image data, making them suitable for extracting and learning features from raw mechanical signals. Reference [16] proposed an improved neural network, MCNN, to extract features from brake friction signals and used SVM to classify these features, achieving the accurate detection of brake faults, indirectly verifying the feasibility of the basic CNN+SVM model. Reference [17] introduced an enhanced convolutional neural network with two expanded receptive fields, improving the model’s feature learning capability and achieving higher accuracy compared to traditional methods in planetary gearbox fault diagnosis. Reference [18] fused multi-source vibration signals collected from multiple sensors into a two-dimensional matrix input to a convolutional neural network, proposing a method for reciprocating compressor fault diagnosis. The literature [19] extracts deep features through a parallel multi-channel structure of 1D-CNN and 2D-CNN and improves fault diagnosis accuracy by employing a feature fusion strategy combined with particle swarm optimization–support vector machine (PSO-SVM). Compared to single-channel models, this model exhibits superior diagnostic performance; however, the use of PSO-SVM and the transformation of one-dimensional data into two-dimensional images increases the research workload and model complexity, and there is a higher risk of information loss during the conversion of one-dimensional data to two-dimensional images.

Currently, algorithms for mechanical equipment fault diagnosis often have a single-structured feature input channel, which limits data interpretability and reduces the capability of feature learning in noisy environments [20]. Moreover, the parameter scale of existing models is often relatively large, requiring substantial computational resources, which leads to lower operational efficiency and makes it difficult to deploy and apply them quickly in industrial settings.

Based on the above analysis, this paper proposes a lightweight and efficient multimodal feature fusion convolutional neural network (LEMFN) for mechanical equipment fault diagnosis. The main contributions of this paper include the following points:

(1) Multimodal and Multiscale Feature Learning: By combining time-domain signals with frequency-domain signals, the model can capture rich fault features at multiple scales, which helps to improve data adaptability across different operating conditions. At the same time, the dual-channel structure design enhances the model’s robustness to noise, making its performance more stable in real industrial environments.

(2) Data Augmentation Techniques: The application of data augmentation methods such as random overlapping sampling technique (ROST) effectively alleviates the risk of overfitting due to limited training samples, promoting a more robust learning process.

(3) Application of Attention Mechanisms: The convolutional block attention module (CBAM) helps the model focus on the most discriminative feature parts, reducing the impact of irrelevant or secondary information on decision-making, thereby further improving the model’s generalization capability.

(4) Adoption of Feature Fusion Strategy: This allows information extracted from two independent paths to complement each other, jointly optimizing the final decision-making process. Meanwhile, the model maintains low computational complexity, making it suitable for resource-constrained practical applications.

The remainder of the paper is organized as follows: Section 2 elaborates on the relevant theoretical foundations; Section 3 describes the proposed method, model, and diagnostic process; Section 4 presents the experimental validation and analysis of the model for fault diagnosis; Section 5 discusses the development and application of the graphical user interface; and Section 6 concludes the work presented in this paper.

## 2. Theoretical Foundations

### 2.1. Convolutional Neural Networks

**Figure 1:**
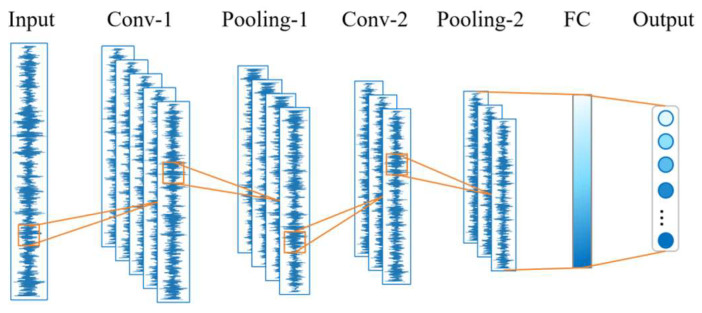
Typical convolutional neural network model.

Convolutional neural networks (CNNs) are feedforward neural networks that perform convolution operations. Their structural characteristics are primarily reflected in the following three aspects: local connections, weight sharing, and pooling. As shown in Figure 1, a typical CNN architecture mainly consists of an input layer, convolutional layers, pooling layers, fully connected layers, and an output layer.

### 2.2. Convolutional Block Attention Module

The convolutional block attention module (CBAM) is a module designed to enhance the performance of convolutional neural networks. It was introduced in 2018 by Sanghyun Woo and colleagues as a self-attention mechanism aimed at improving the network’s ability to focus on important features, thereby enhancing the model’s accuracy and robustness. Traditional convolutional neural networks primarily rely on convolutional layers to extract local features from images, followed by feature integration through pooling or fully connected layers. However, such structures may not effectively capture global contextual information or inter-channel dependencies. CBAM introduces an attention mechanism that allows the network to dynamically adjust its focus, emphasizing more relevant feature regions and channels, thus improving the model’s performance.

**Figure 2:**
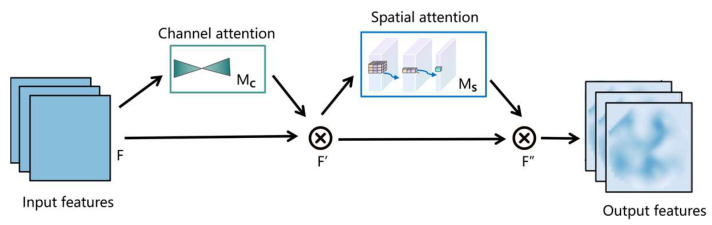
Structure of the convolutional block attention module.

CBAM consists of two attention sub-modules: channel attention (CA) and spatial attention (SA).Channel attention (CA): This focuses on the importance of different feature information or channels. It first calculates a global descriptor for each channel, then generates weights for each channel using a multi-layer perceptron (MLP). These weights are subsequently used to weight the feature information, allowing the network to pay more attention to the more significant feature information. Spatial attention (SA): This focuses on which locations within the feature information are more important. After computing the channel attention, it highlights the important positional information within the feature information. By learning the importance weights for each channel and each spatial position, and applying these weights to the features, the useful information is enhanced while the less useful information is suppressed [21]. The structure of CBAM is illustrated in Figure 2.

### 2.3. Data Processing and Transformation

#### Random Overlapping Segmentation

Currently, sample segmentation generally adopts two methods, namely non-overlapping segmentation and overlapping segmentation. Non-overlapping segmentation, due to its fixed cutting positions, might lead to the loss of some sample fault information, and when the original signal length is relatively short, non-overlapping segmentation produces fewer samples. In contrast, the overlapping segmentation approach can avoid the loss of sample information to a certain extent and also generate more samples, which is considered a method of data augmentation [22]. Although the overlapping segmentation method can produce more samples by increasing the overlap ratio, once the overlap ratio is determined, the position of each segmented sample within the original signal segment is fixed, still resulting in the insufficient utilization of continuous signal segments’ information.

**Figure 3:**
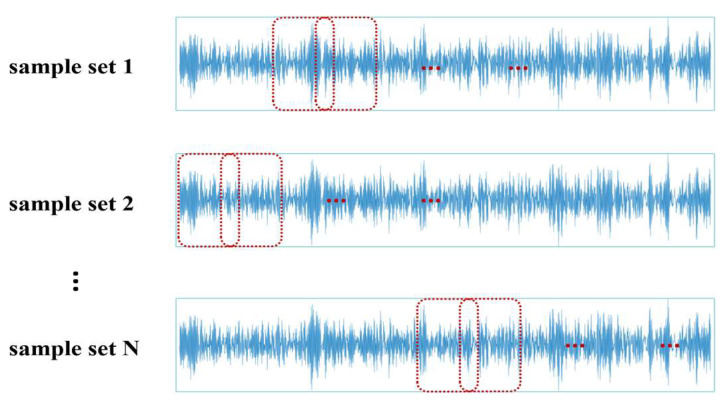
Schematic diagram of random overlapping segmentation of sample sets.

To further utilize the information from the original signal data and simulate the randomness of signals when faults occur, this paper adopts a signal segmentation method called random overlapping segmentation (ROS). The term “random” refers to the fact that the cutting positions of the samples are randomly selected; and “overlapping segmentation” means that adjacent samples share a portion of the same data points. By using random overlapping segmentation to construct the sample set, different batches of samples are generated each time, as shown in Figure 3. With sufficient training, theoretically, any position of the signal segment can be segmented, leading to better data augmentation effects and consequently enhancing the generalization ability of the diagnostic model.

## 3. Model Structure and Diagnostic Process of the Proposed Method

### 3.1. Model Structure Description

**Figure 4:**
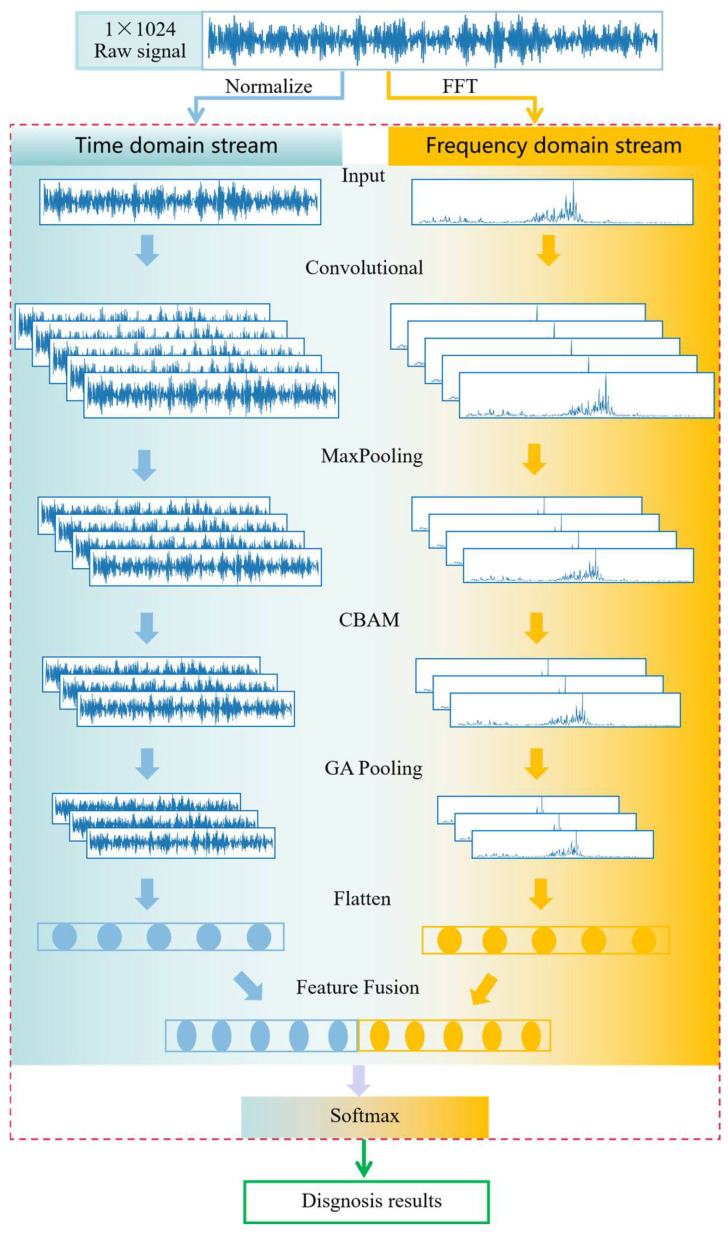
Proposed model structure.

The architecture of the proposed lightweight and efficient multimodal feature fusion convolutional neural network (LEMFN) for mechanical equipment fault diagnosis is shown in Figure 4. The network consists of the following two parallel parts: a one-dimensional time-domain data stream CNN channel and a one-dimensional frequency-domain data stream CNN channel. This allows for simultaneous feature extraction from both time-domain and frequency-domain data, making the use of data more comprehensive and the extracted fault information more thorough. The entire network structure comprises an input layer, convolutional layers, pooling layers, a convolutional attention mechanism layer, a global average pooling layer, a feature flattening layer, a feature fusion layer, and an output layer.

### 3.2. Specific Structure and Parameters of the Network

The specific structure of the network and the configuration of parameters for each layer are shown in Table 1.

This model is a dual-channel convolutional neural network architecture that combines raw time-domain vibration signals and frequency-domain signals. This architecture can extract fault features at multiple scales, thereby improving the accuracy of fault pattern recognition. The time-domain channel processes the raw vibration signals directly, capturing local features in the time domain; the frequency-domain channel handles the frequency-domain signals after Fast Fourier Transform (FFT), revealing the time–frequency distribution information of the signal at different frequencies. The main characteristics are as follows:

(1) Multimodal and Multiscale Feature Extraction: By combining the raw vibration signals from the time-domain channel with the FFT signals from the frequency-domain channel, the model can extract fault features at multiple scales, allowing for a more comprehensive utilization of fault information.

(2) Complementary Information Fusion: The time-domain channel captures local features in the time domain, while the frequency-domain channel reveals the time–frequency distribution information of the signal at different frequencies. The fusion of these two types of information enables the model to recognize fault patterns more accurately.

(3) Enhanced Focus on Key Features: The CBAM, through its channel attention and spatial attention sub-modules, automatically weights important feature regions, enhancing the model’s focus on key fault information. This helps the model better capture fault features and improve diagnostic performance.

(4) Increased Training Sample Diversity: The random overlapping sampling technique (ROST) generates more training samples by randomly selecting overlapping data segments, which helps to alleviate the problem of data scarcity. Additionally, the diversified training samples enable the model to better adapt to different operating conditions, thereby improving its generalization ability.

(5) Simplified Network Structure: A global average pooling layer replaces the fully connected layer, reducing the number of parameters and simplifying the network structure. Reducing the parameter count helps to lower the risk of overfitting and enhances the robustness of the model.

### 3.3. Diagnosis Process

**Figure 5:**
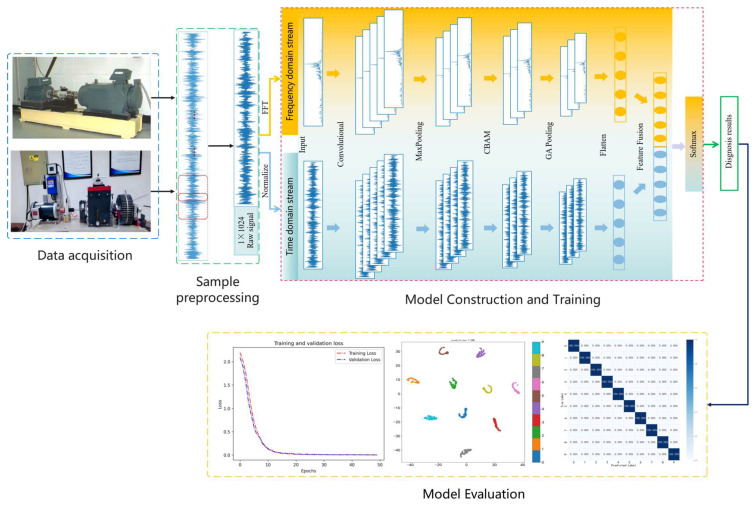
LEMFN fault diagnosis flowchart.

The proposed fault transfer diagnosis method mainly includes the following four parts: data acquisition, sample preprocessing, model building and training, and model evaluation. The procedural framework of the method in this paper is illustrated in Figure 5, and the main steps are as follows:

(1) Signal Acquisition: Vibration signals corresponding to various fault states of the mechanical equipment are collected using accelerometers on two different test benches. These signals serve as input samples for the fault diagnosis model.

(2) Sample Preprocessing: The acquired fault signals are augmented using random overlapping sampling technology. Then, the one-dimensional time-domain signals are transformed into one-dimensional frequency signals using the Fast Fourier Transform (FFT) method [23,24,25]. The samples are divided into training and testing sets in a 7:3 ratio.

(3) Model Building and Training: After constructing the proposed model structure and configuring the parameters, and dividing the training and testing datasets, the next step is to set up the model training parameters, including the training learning rate, loss function, and batch size for training. During the training process, the learning rate is set to 0.001, and the Adam optimizer is used for parameter updates. Additionally, the loss function chosen is the categorical cross-entropy loss function; the batch size for input samples at each training step is 64. After each model training session, the model’s diagnostic accuracy is tested using the test set to further validate the diagnostic capability of the model until the model reaches optimal network performance.

(4) Model Evaluation: The model is thoroughly evaluated using metrics such as diagnostic classification accuracy, loss function values, confusion matrices, t-distributed Stochastic Neighbor Embedding (t-SNE), recall, and F1 score, to examine the model’s ability to learn and understand fault features.

## 4. Fault Diagnosis Experiment Validation and Analysis

### 4.1. Experiment One

#### 4.1.1. Description of Dataset I

The bearing dataset from Case Western Reserve University (CWRU) [26,27] in the United States is one of the most widely used open-source datasets for mechanical equipment fault diagnosis. To enhance the reference value of our work, we chose to conduct experiments on this publicly available dataset.

**Figure 6:**
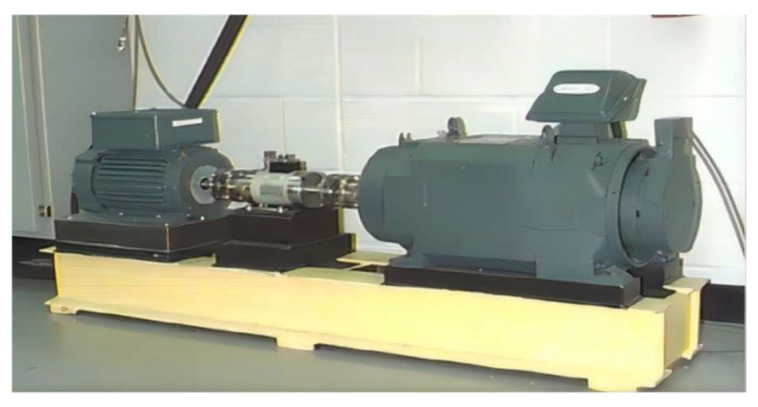
CWRU bearing fault diagnosis experimental platform.

The experimental setup for the CWRU bearing dataset is shown in Figure 6, featuring SKF-6205 drive-end bearings and SKF-6203 fan-end bearings. For this experiment, we selected the drive-end bearing with a sampling frequency of 12 kHz and included 10 fault states. Each experiment uses 100 samples per fault category, totaling 1000 samples, with each individual sample containing 1024 data points. The samples are divided into training and testing sets at a ratio of 7:3. The specific details are provided in Table 2.

**Figure 7:**
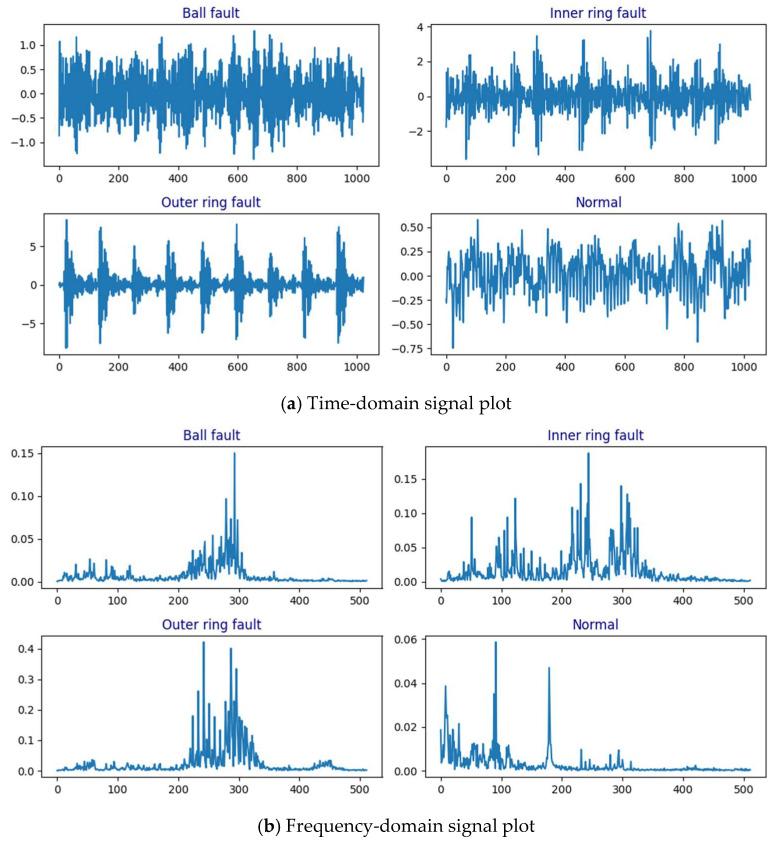
Time- and frequency-domain signals of the CWRU dataset.

The time-domain signals and frequency-domain signals obtained through Fast Fourier Transform (FFT) for inner race fault (IR), ball fault (B), outer race fault (OR), and normal condition are shown in Figure 7a,b.

#### 4.1.2. Diagnosis Results and Analysis

♦ (1) Analysis of Diagnostic Accuracy and Loss Function Values

TDSCN and FDSCN are two independent networks consistent with the time-domain stream and frequency-domain stream channels of the LEMFN model, respectively; TDSCN* and FDSCN* are the TDSCN and FDSCN networks without introducing CBAM; LEMFN* is the LEMFN model network without introducing CBAM.

**Figure 8:**
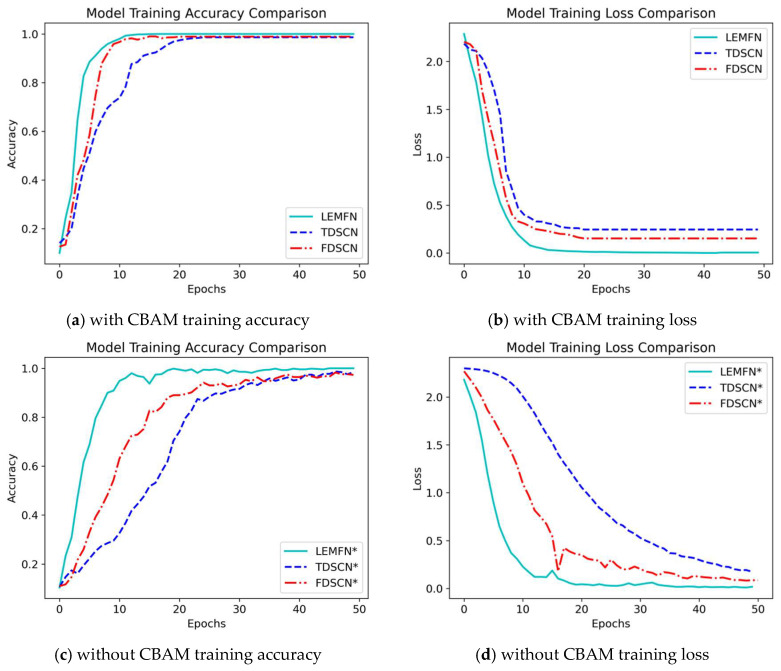
Loss function values and diagnostic accuracy.

Figure 8 shows the changes in accuracy values and loss values during the training process. As can be seen from Figure 8a–d, after introducing CBAM into each model, their convergence speed and diagnostic stability have been improved to a certain extent, which demonstrates the effectiveness of CBAM. At the same time, it can be observed that the performance of LEMFN and LEMFN* is better than that of their sub-models in both channels, which proves the effectiveness of the feature fusion strategy, enabling the model to extract fault features at multiple scales, thus improving the accuracy and stability of fault pattern recognition.

♦ (2) Analysis of t-SNE Results

**Figure 9:**
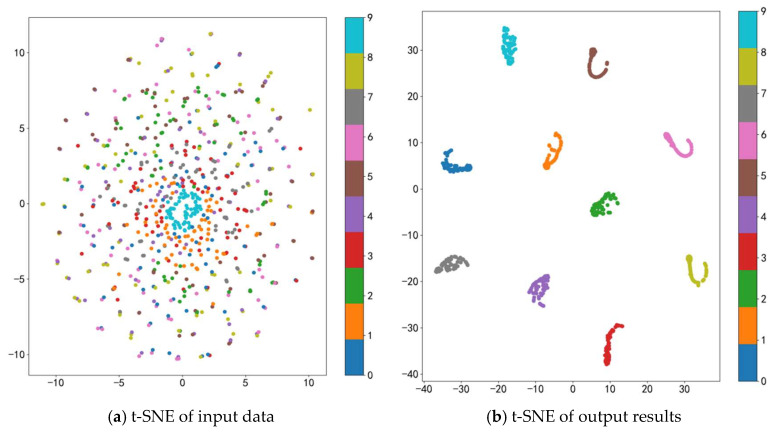
Feature visualization.

To verify the feature extraction capability of the proposed method, the t-distributed Stochastic Neighbor Embedding (t-SNE) algorithm is utilized to reduce the high-dimensional data from the input and output layers to two dimensions for visualization. The clustering results of the original data and the feature data extracted by the proposed model are shown in Figure 9. From Figure 9a, it can be observed that the original input data are highly mixed in the two-dimensional space, indicating a high degree of confusion among different types of data. From Figure 9b, it can be seen that after the model extracts and learns the input signal data, the data corresponding to different health states are well grouped. In the output layer, the intra-class compactness and inter-class separability of the health state features are significantly enhanced, the boundaries between different health states become clear and distinct, data points within the same class are more tightly clustered, and the confusion between different features is eliminated. This indicates that the proposed method can effectively extract and learn the features of different health states in the bearing data and accurately identify the corresponding fault types.

♦ (3) Analysis of Confusion Matrix Results

**Figure 10:**
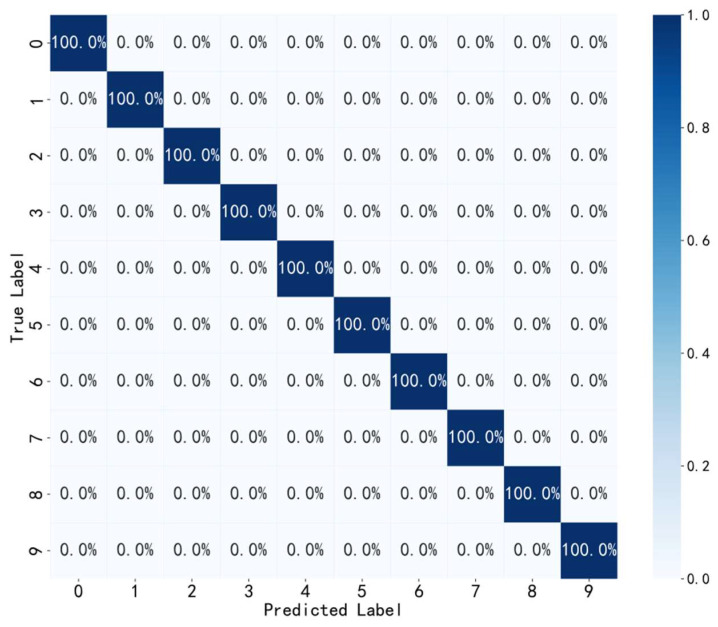
Confusion matrix.

To further illustrate the effectiveness and classification performance of the proposed method, a confusion matrix analysis is conducted on one of the test results from the dataset. Figure 10 shows the visualization of the classification results of the test set using the confusion matrix. It can be seen that all predictions are entirely correct, thus achieving a diagnostic accuracy of 100% in the final diagnosis results, which is consistent with the results shown in Figure 8. This further demonstrates that the model possesses excellent fault diagnosis classification performance.

♦ (4) Robustness Analysis

**Figure 11:**
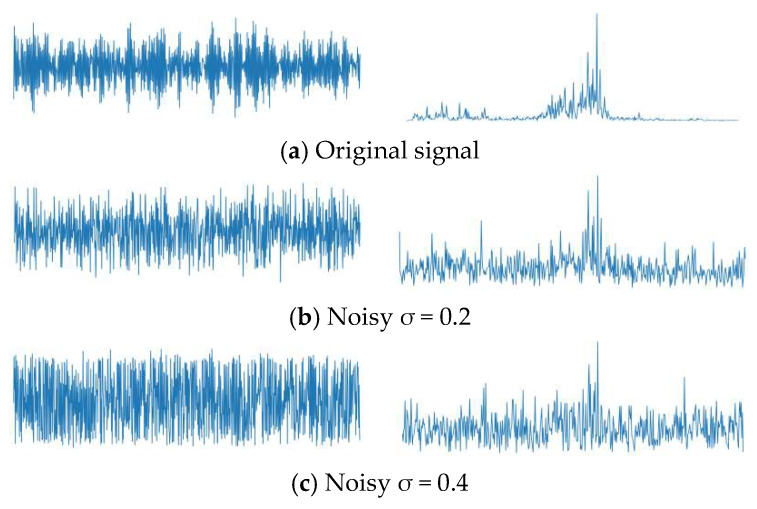
Time- and frequency-domain signals after adding Gaussian noise.

In this study, we utilized 2 horsepower (HP) data and added Gaussian white noise with standard deviations of 0.2 and 0.4, respectively, to simulate the noise interference that bearings may encounter during operation. The time-domain and frequency-domain plots of the data after adding the noise are shown in Figure 11.

**Figure 12:**
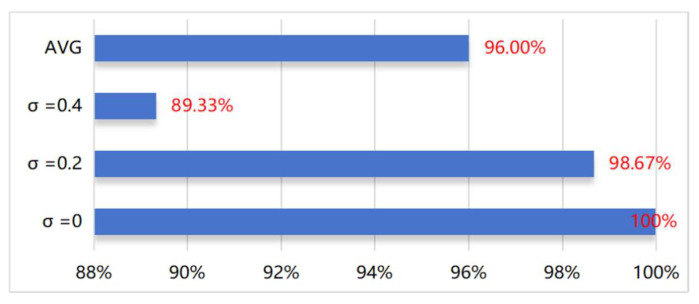
Diagnostic accuracy of the model under different noise levels.

Subsequently, these noisy signals were fed into the network as training data, and tests were conducted, with the diagnostic accuracy of the test results presented in Figure 12. This process aims to evaluate the network’s adaptability and diagnostic performance under different levels of noise.

From the results in Figure 12, it can be seen that compared to the original unnoised data, the addition of varying levels of extra Gaussian white noise led to different changes in the model’s diagnostic accuracy. From Figure 11, it can be observed that when σ = 0.2 Gaussian noise is added, the signal features compared to the original signal are disturbed and masked by the noise but still retain most of the valid features; however, when σ = 0.4 Gaussian noise is added, the relevant discriminative information in the signal is severely disturbed and masked, significantly increasing the difficulty of diagnosis for all methods.

When σ = 0.2 Gaussian noise is added, the proposed model (LEMFN) still achieves a very high result, with classification accuracy of 98.67%. When σ = 0.4 Gaussian noise is added, there is a significant decrease in the model’s classification accuracy, with a classification accuracy of 89.33%, yet still achieving a relatively high result. The above analysis indicates that the LEMFN model has good robustness against additional noise, showing better classification capability.

♦ (5) Generalization Performance Analysis

The operational state of mechanical equipment is complex and variable, and the features of the measured signals exhibit significant differences. To verify the ability of the proposed model (LEMFN) to identify bearing faults under varying operating conditions, a generalization performance experiment was conducted.

**Figure 13:**
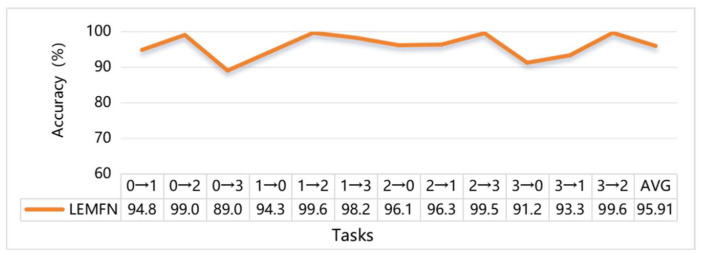
Test tasks and diagnostic accuracy.

Let 0, 1, 2, and 3 represent motor loads of 0 HP, 1 HP, 2 HP, and 3 HP, respectively, corresponding to four operating conditions. In the figure, 0→1 indicates that the dataset from condition 0 is used to train the model, while the dataset from condition 1 is used for testing. The specific task settings and diagnostic results are shown in Figure 13.

It can be observed that across twelve diagnostic tasks, the proposed model (LEMFN) provided good classification results, with an average diagnostic accuracy of 95.9%. Furthermore, analyzing individual tasks reveals that the model achieved relatively lower diagnostic accuracy in tasks 0→3, 3→0, and 3→1. This is because the load difference between these two conditions is large, making the conditions more complex; hence, the diagnostic performance is comparatively worse than other conditions, which aligns with reality. This also indicates that the proposed multimodal feature fusion parallel time-frequency dual-stream method, by leveraging fused time-domain and frequency-domain information, effectively reduces the distribution differences between data domains under different operating conditions, achieving better generalization ability and classification performance.

♦ (6) Analysis of Model Diagnostic Accuracy, Parameter Count, and Single-Step Computation Time

As shown in Table 3, we compare the diagnostic accuracy, parameter count, and single-step computation time of the LEMFN model with MobileNet [28] (a lightweight convolutional neural network architecture designed for mobile and embedded devices) and Vision Transformer [29] (ViT, a significant model representative of the Transformer architecture).

From the table, it can be seen that the proposed LEMFN model has better advantages in terms of diagnostic accuracy, parameter count, and single-step computation time, followed by MobileNet, while ViT performs the worst overall. Although the ViT possesses long-range modeling and parallel computing capabilities, achieving good performance typically requires a large number of data samples, which is difficult to achieve in the field of fault diagnosis. Additionally, due to its self-attention mechanism, it contains a large number of parameters, leading to higher computational requirements. As a lightweight convolutional neural network architecture specifically designed for mobile and embedded devices, MobileNet has a significantly smaller parameter count compared to ViT. While Transformer models have achieved great success in natural language processing and image domains, CNNs are more advantageous in the application of mechanical equipment fault diagnosis.

The above analysis shows that the LEMFN model maintains high diagnostic accuracy, strong robustness, and generalization capability (as illustrated in Figure 12 and Figure 13), while having low computational complexity and reasonable hardware requirements. This model can run efficiently on common workstation configurations when deployed, making it suitable for industrial field applications.

### 4.2. Experiment Two

#### 4.2.1. Description of Dataset II

**Figure 14:**
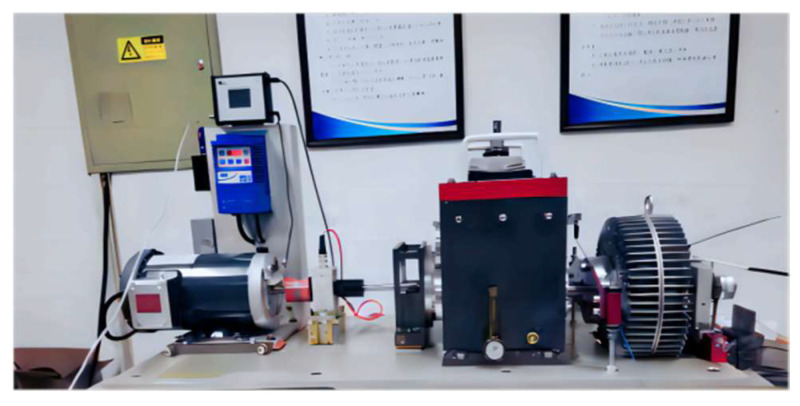
Rotating machinery fault simulation test platform.

The dataset used for this experiment, referred to as Dataset II, was collected from a rotating machinery fault simulation test platform, as shown in Figure 14. The experiment tested three different health conditions: bearing fault (ball fault), rotor bending fault, and normal conditions. The mechanical vibration signals were collected at a sampling frequency of 20.48 kHz, with a load of 0HP and a rotational speed of 1500 r/min. Each operational state’s data were segmented into samples of 1024 sampling points. The training set and testing set were divided in a 7:3 ratio, as detailed in Table 4.

#### 4.2.2. Diagnosis Results and Analysis

♦ (1) Model Precision, Recall, F1 Score, and Accuracy Metrics

Precision, recall, F1 score, and accuracy were selected as evaluation metrics to analyze the diagnostic performance of the model. These metrics are crucial references for evaluating the performance of an AI model. As shown in Table 5, the proposed model achieves up to 100% in all metrics, indicating that the model possesses excellent diagnostic classification performance and significant application potential.

♦ (2) Scatter Plot of Predicted vs. True Values

**Figure 15:**
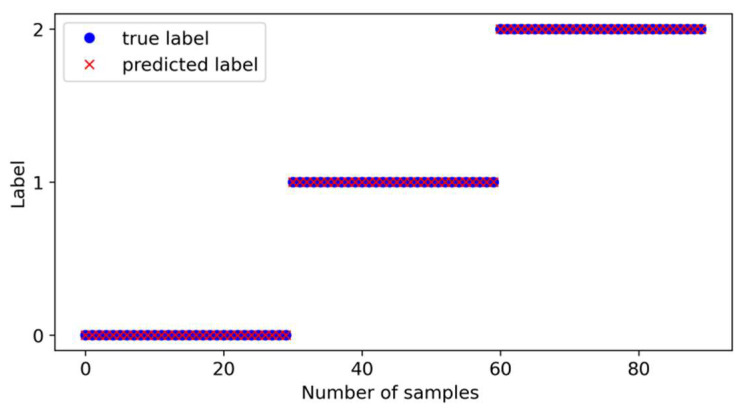
Scatter plot of predicted values vs. true values.

The experimental verification results are shown in Figure 15. The model’s prediction results for various health states are consistent with the actual health state types, and all predictions are correct, further demonstrating the superior diagnostic classification ability and stability of the proposed model.

### 4.3. Comparison of Diagnostic Accuracy with Other State-of-the-Art Algorithm Models

**Figure 16:**
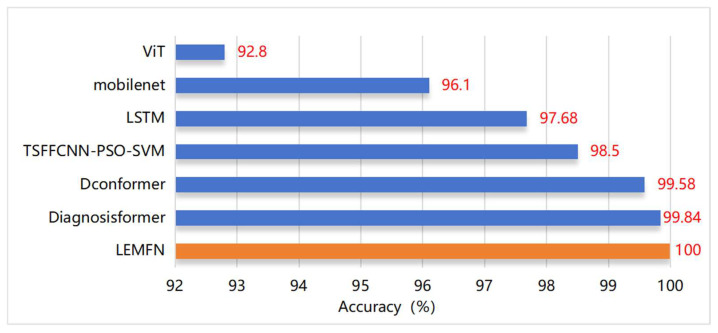
Comparison of model diagnostic accuracy.

As shown in Figure 16, we compare the diagnostic accuracy results of the LEMFN model proposed in this paper with other state-of-the-art algorithm models, including MobileNet, ViT, LSTM, TSFFCNN-PSO-SVM, Dconformer [30], and Diagnosisformer [31].

From Figure 16, it can be observed that among the six other algorithms compared with the proposed model, the ViT model has the lowest diagnostic accuracy, while the Diagnosisformer, which is an improved Transformer-based model, has the highest, with accuracies of 92.8% and 99.84%, respectively. The TSFFCNN-PSO-SVM, which also adopts a parallel multi-channel structure, shows better diagnostic performance compared to the single-channel ViT, MobileNet, and LSTM. The diagnostic accuracy of the LEMFN model proposed in this paper is the highest among all the models, improving by 1.5% over the TSFFCNN-PSO-SVM and still showing an improvement over the Diagnosisformer. This indicates that the proposed model is better at extracting and learning the fault feature information of mechanical equipment, resulting in superior diagnostic classification capabilities.

## 5. Development and Application of the Graphical User Interface

### 5.1. System Login Interface

**Figure 17:**
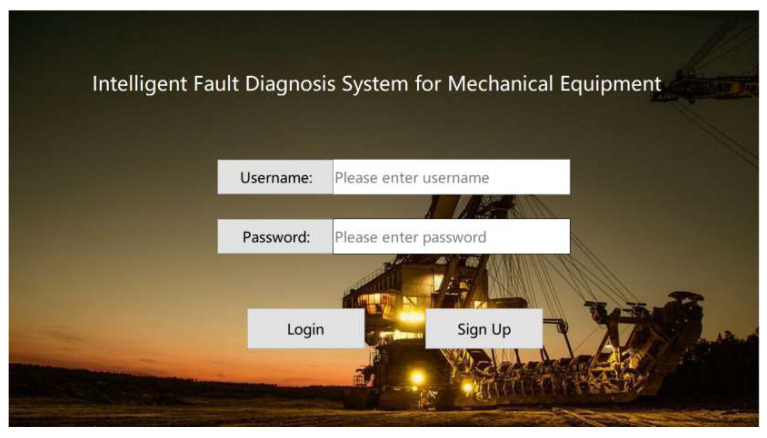
Login interface of the diagnostic system.

To ensure data security and manage permissions, the system requires the input of an account and password for login. The login interface is shown in Figure 17.

### 5.2. Main Interface of the Diagnostic System

**Figure 18:**
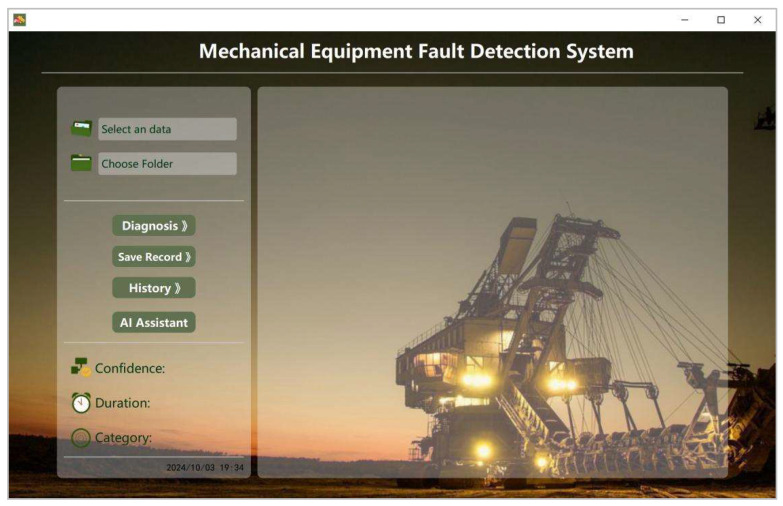
Main interface of the diagnostic system.

After entering the correct account and password, the user is directed to the main interface of the mechanical equipment fault diagnosis system, as shown in Figure 18. The main interface includes three functional parts, as follows: data access and reading, intelligent diagnosis, and diagnostic result monitoring. Below is an introduction to these three parts and their implementation methods.

(1) Data Access and Reading

The system offers two modes for data access and reading. The first mode involves selecting a single piece of data to achieve a one-time fault diagnosis; the second mode involves accessing and reading data in folder form to enable batch detection and identification of faults.

(2) Intelligent Diagnosis and Monitoring of Diagnostic Results

**Figure 19:**
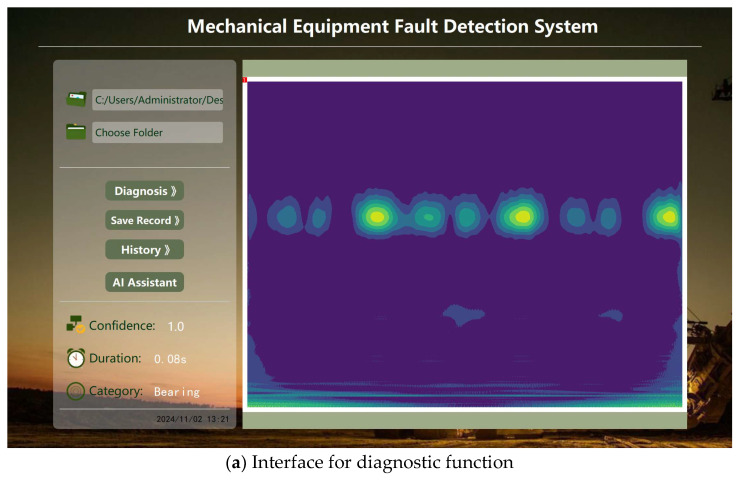

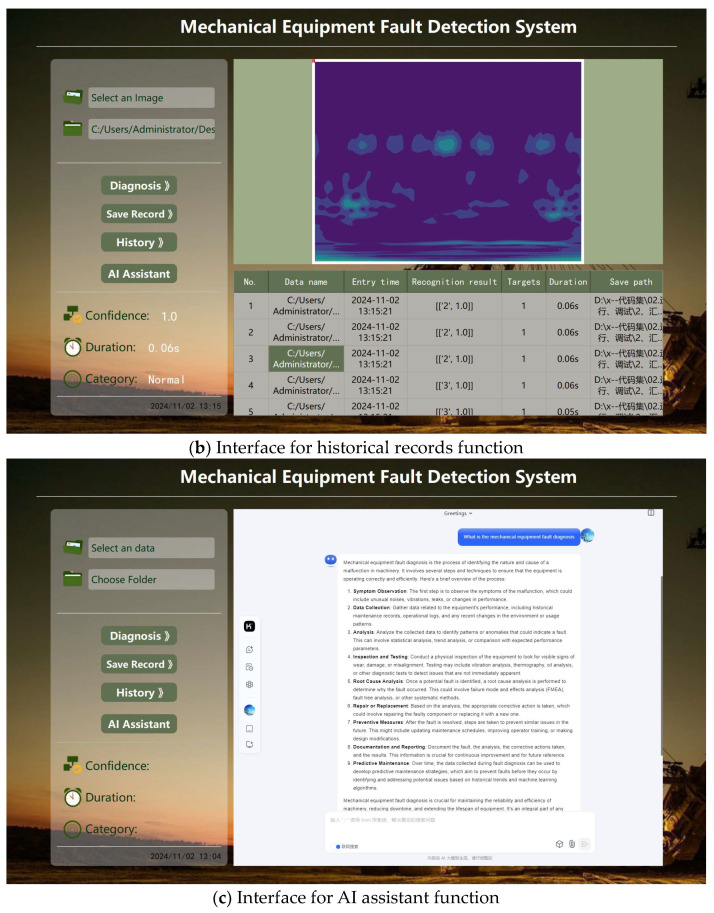
Demonstration of main functional interfaces.

Once data are loaded into the system, fault detection and recognition are carried out. As shown in Figure 19a, the time-frequency graph corresponding to the loaded data are displayed on the right side, while the diagnostic result, processing time, and confidence level are shown at the lower left.

As illustrated in Figure 19b, the system can record inspected data and allows for reviewing historical inspection results individually. Additionally, the system provides functionality to export recorded inspection results, facilitating the information management of inspection outcomes.

To enhance work efficiency, an AI assistant feature has also been embedded in the system, as depicted in Figure 19c. It is capable of utilizing powerful AI models like ChatGPT and Kimi to offer assistance.

## 6. Conclusions

To address issues such as single-feature input channel structures, incomplete data interpretation, insufficient feature learning capabilities in noisy environments, and large model parameters that hinder application in actual industrial scenarios with limited computing resources, this paper proposes a lightweight and efficient multimodal feature fusion convolutional neural network (LEMFN). The main conclusions of this study are summarized as follows:

(1) Multimodal multiscale feature learning: By integrating time-domain signals and frequency-domain signals, LEMFN can capture rich fault features at different scales, which helps enhance the model’s data adaptability under various operating conditions. Meanwhile, the dual-channel structure design improves the model’s robustness to noise.

(2) Data augmentation techniques: The effective application of ROST significantly alleviates overfitting due to limited training samples, fostering a more robust learning process.

(3) Application of attention mechanisms: The introduction of CBAM assists the model in focusing on the most discriminative feature parts, reducing the impact of irrelevant or secondary information on final decision-making, thereby further enhancing the model’s performance.

(4) Computational efficiency and model lightness: LEMFN boasts high diagnostic accuracy and rapid convergence capabilities while maintaining low computational complexity, making the model suitable for deployment in real-world application environments with constrained computing resources.

(5) Development of a graphical user interface: The development of this system contributes to advancing the practical application of intelligent fault diagnosis in mechanical equipment.

## Figures and Tables

**Table 1:** Specific structure and parameter configuration of the LEMFN model.

Layer Names and Parameter Configuration			
Time-domain stream		Frequency-domain stream	
1D_Input layer	(64, 1 × 1024, 1)	1D_Input layer	(64, 1 × 512, 1)
1D_Conv layer1-1	(32, 8, 1, relu)	1D_Conv layer 1-1	(32, 8, 1, relu)
1D_MaxPooling layer 2-1	(4, 2)	1D_MaxPooling layer 2-1	(4, 2)
1D_Conv layer 1-2	(64, 8, 1, relu)	1D_Conv layer 1-2	(64, 8, 1, relu)
1D_MaxPooling layer 2-2	(4, 2)	1D_MaxPooling layer 2-2	(4, 2)
1D_Conv layer 1-3	(128, 4, 1, relu)	1D_Conv layer 1-3	(128, 4, 1, relu)
1D_CBAM layer	SA_Conv:(1, 5, sigmoid, padding = “same”)	1D_CBAM layer	SA_Conv:(1, 5, sigmoid, padding = “same”)
1D_GlobalAveragePooling layer	—	1D_GlobalAveragePooling layer	—
Flatten layer	Output size = (64, 128)	Flatten layer	Output size = (64, 128)
Feature Fusion and Classification			
Feature Fusion layer		Output size = (64, 256)	
Output layer (Softmax)		Output size = (64, 10)	

**Table 2:** Description of bearing experiment data.

Label	Status	Fault Sizes (mm)	Training Set	Testing Set	Sample Lengths
0	B007	0.1778	70	30	1024
1	B014	0.3556	70	30	1024
2	B021	0.5334	70	30	1024
3	IR007	0.1778	70	30	1024
4	IR014	0.3556	70	30	1024
5	IR021	0.5334	70	30	1024
6	OR007	0.1778	70	30	1024
7	OR014	0.3556	70	30	1024
8	OR021	0.5334	70	30	1024
9	Normal	—	70	30	1024

**Table 3:** Comparative analysis of model performance.

Models	Accuracy	Parameters	Single Step Duration
LEMFN	100.0%	0.40 M	0.0653 S
mobilenet	96.1%	5.40 M	0.0678 S
ViT	92.8%	86.57 M	100.6836 S

**Table 4:** Description of bearing experiment data.

Label	Status	Training Set	Testing Set	Sample Lengths
0	Bearing fault	70	30	1024
1	Rotor fault	70	30	1024
2	Normal	70	30	1024

**Table 5:** Diagnostic performance of the proposed model.

Status	Precision	Recall	f1-Score	Support
Bearing fault	1.00	1.00	1.00	10
Rotor fault	1.00	1.00	1.00	10
Normal	1.00	1.00	1.00	10
accuracy	-	-	1.00	30
macro avg	1.00	1.00	1.00	30
weighted avg	1.00	1.00	1.00	30

## Data Availability

Data can be made fully available upon request.
